# The impact of pre-operative angiography on nephron-sparing surgery and outcome in wilms tumor arising from horseshoe kidney: case report and literature review

**DOI:** 10.3389/fsurg.2025.1528438

**Published:** 2025-04-08

**Authors:** Angelo Zarfati, Giovanni Rollo, Giulia Cassanelli, Chiara Grimaldi, Giorgio Persano, Gian Luigi Natali, Annalisa Serra, Silvia Madafferi, Alessandro Inserra, Cristina Martucci, Alessandro Crocoli

**Affiliations:** ^1^ General and Thoracic Surgery Unit, Bambino Gesù Children’s Hospital, IRCCS, Rome, Italy; ^2^ Department of Systems Medicine, University of Tor Vergata, Rome, Italy; ^3^ Surgical Oncology Unit, Bambino Gesù Children’s Hospital, IRCCS, Rome, Italy; ^4^ Interventional Radiology Unit, Bambino Gesù Children’s Hospital, IRCCS, Rome, Italy; ^5^ Hematology/Oncology, Cell Therapy, Gene Therapies and Hemopoietic Transplant, Bambino Gesù Children’s Hospital, IRCCS, Rome, Italy

**Keywords:** nephroblastoma, wilms, horseshoe kidney, nephron sparing, angiography

## Abstract

**Background:**

The horseshoe kidney (HSK) is a congenital renal anomaly characterized by the fusion of two distinct units, which are connected by a parenchymal bridge. HSK has a terminal vascular supply as a normal kidney. In the uncommon event that a Wilms Tumor (WT) (or Nephroblastoma) results from an HSK, Nephron-Sparing Surgery (NSS) is required. Since the anatomy expressed by HSK can vary substantially, an extensive preoperative evaluation of the vascularization is required to increase the likelihood of NSS and reduce complications. However, the role of pre-operative angiography (PORA) in this setting has not yet been defined. Our aim was to define the impact of PORA on NSS and its outcomes in WT arising from HSK.

**Case presentation:**

We presented a case of an 8-year-old girl with a WT in a HSK, for whom the PORA was safe and helpful in terms of NSS and surgical outcomes. Additionally, we performed a review of the literature regarding PORA's effects on the surgical and clinical outcomes of WT in patients with HSK.

**Conclusion:**

PORA seemed potentially safe and useful in terms of NSS and complications. Despite its invasiveness, this interventional study may have a role in the extremely selected group of patients with WT arising from an HSK. Further studies are needed to validate our results.

## 1 Introduction

Nearly 90% of pediatric renal tumors are caused by Wilms tumor (WT) or nephroblastoma (1). Horseshoe kidney (HSK) is a relatively common fusion renal anomaly consisting in two units connected by a bridge of functional tissue (2). The risk of WT is doubled for patients with HSK (3). The rare diagnosis of WT arising from a HSK mandates a Nephron-Sparing Surgery (NSS) (1, 4). NSS consists in the complete resection of the tumor while preserving as much of parenchyma as possible (5).

A detailed preoperative assessment of vascularization is required due to the fact that HSK may exhibit extremely variable anatomy (2, 4, 6). Given the absence of a definition of NSS in pediatric HSK, we have recently developed and proposed a new definition to address this gap (7).

The available evidence concerning the optimal diagnostic and therapeutic strategies in this uncommon context is extremely scarce. No specific recommendation nor consensus is present (1). Multimodal imaging is most likely conducted by non-invasive methods (MRI/CT) (4, 8). The role of pre-operative renal angiography (PORA) in this context may be beneficial; however, its specific function in pediatric populations remains inadequately defined (3, 9–14).

The aim of the study was to describe our experience with PORA in the case of a WT arising from a HSK and to evaluate its impact on surgical outcome in terms of NSS and complications. Additionally, we conducted a review of the existing literature to contextualize our experience.

## 2 Case presentation

An 8-year-old girl was referred to our tertiary center for an abdominal mass diagnosed nearly 2 years earlier at a local institution. The lesion was initially classified as a “right adrenal asymptomatic mass”. Adrenocorticotropic hormone and urinary catecholamines were normal. A biopsy was not performed. The child was monitored through regular clinical and radiological evaluations.

After nearly two years of uneventful follow-up, the patient was referred to our institution. The physical examination revealed an Non-painful, firm, palpable mass in the right flank/lumbar fossa. The US confirmed that the lesion came from an unrecognized HSK. No additional congenital anomalies were reported. CT showed a 95 × 89 × 135 mm lesion arising from the right half of the HSK. The mass was hypodense and well vascularized. The child presented with multiple bilateral lung lesions. A percutaneous US-guided biopsy of the abdominal mass was performed, confirming the diagnosis of nephroblastoma (blastematous prevalence >90%, Ki67 80%), with no evidence ofanaplasia.

**Figure 1:**
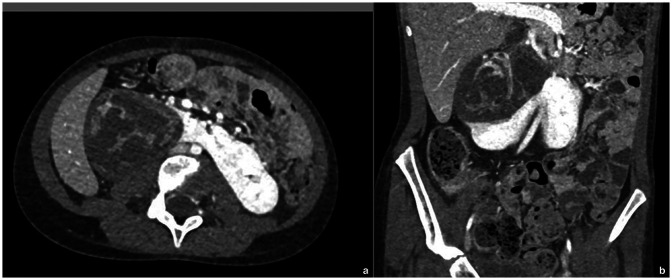
Volumetric decrease of the size of the tumor in an enhanced CT scan. **(a)** Axial contrast enhanced abdomen CT scan (venous phase): volumetric decrease of the solid tumor arising from horseshoe kidney’ side (6.8 × 5.2 × 6.6 cm vs. 13.5 × 8 × 11 cm) after neo-adjuvant chemotherapy. Hypodense intralesional areas show necrotic effect after treatment. **(b)** Coronal contrast-enhanced abdomen CT scan (venous phase): volumetric decrease of the mass shows a decreased mass effect on the IVC and the abdominal aorta but residual compression on the remnant kidney parenchyma.

The child was stage IV at diagnosis. According to the UMBRELLA RTSG-SIOP 2016 protocol, neoadjuvant chemotherapy was started. After six weeks of chemotherapy (vincristine, actinomycin-D doxorubicin), The primary tumor exhibited a reduction in size (68 × 52 × 66 mm), accompanied by a complete regression of the lung metastases (Figure 1).

The case was discussed in a multidisciplinary meeting, and the resection of the lesion was recommended.

**Figure 2:**
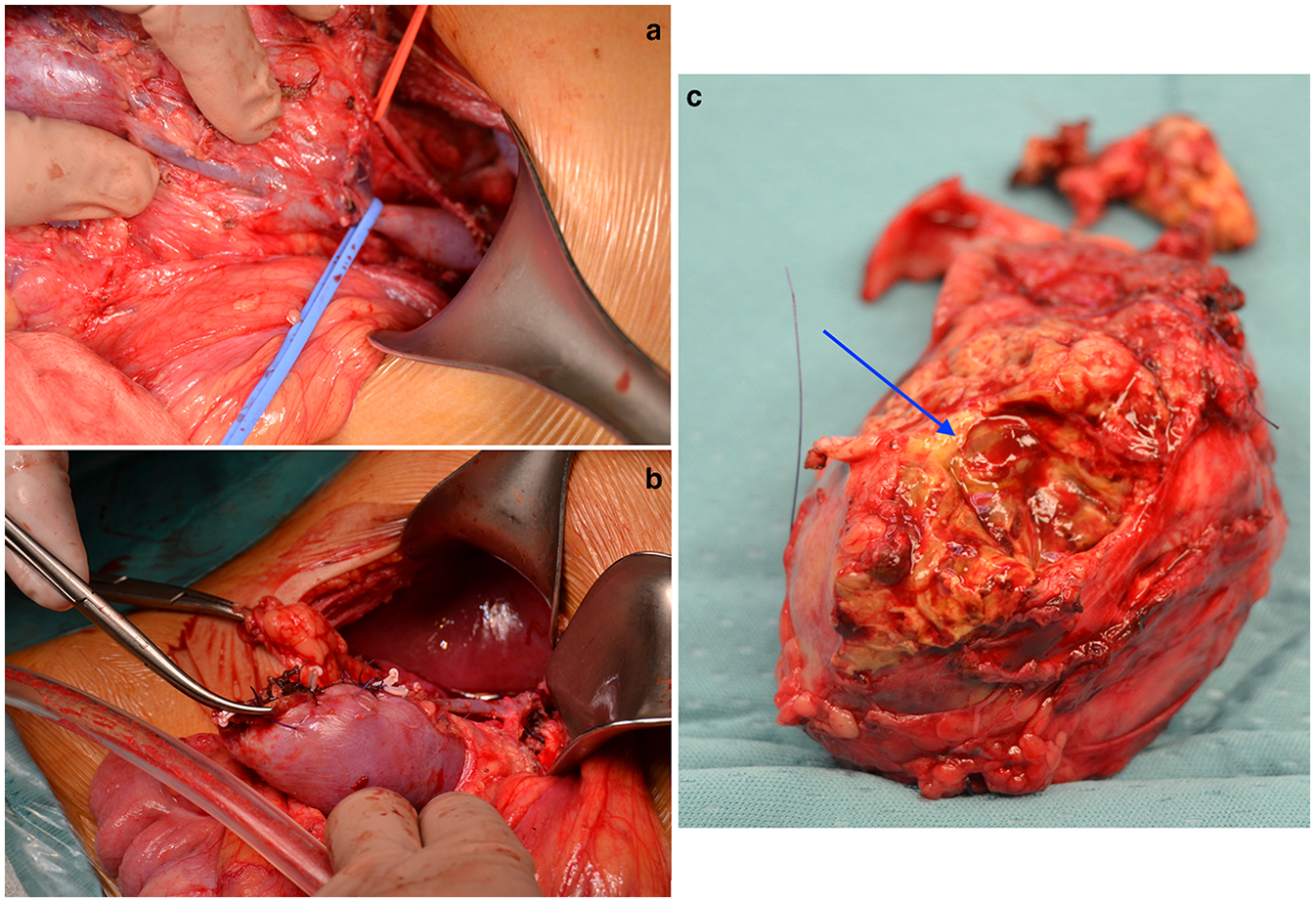
Intraoperative aspect of the WT arising from the HSK. **(a)** Intraoperative image showing the vascular features of the tumor: the right renal artery (on the red vessel loop) and vein (on the blue vessel loop). **(b)** Intraoperative image showing the reconstruction technique performed on the remaining renal parenchyma, via vicryl separate sutures and hemolock used as pledgets. **(c)** Tumoral specimen after the procedure. The blue arrow indicates the area of surgical excision.

PORA was conducted to asses arterial anatomy. Aortography, renal selective, and intrarenal super-selective angiographic runs were conducted via a 4-Fr transfemoral arterial access, revealing vascular afferences to the tumor and HSK (Figure 2). The procedure revealed a single renal artery on the right side and two renal arteries on the left side. An independent arterial branch to the mass was observed on the right, located caudally to the right renal artery. No complications were observed. The post-procedural course was stable.

**Figure 3:**
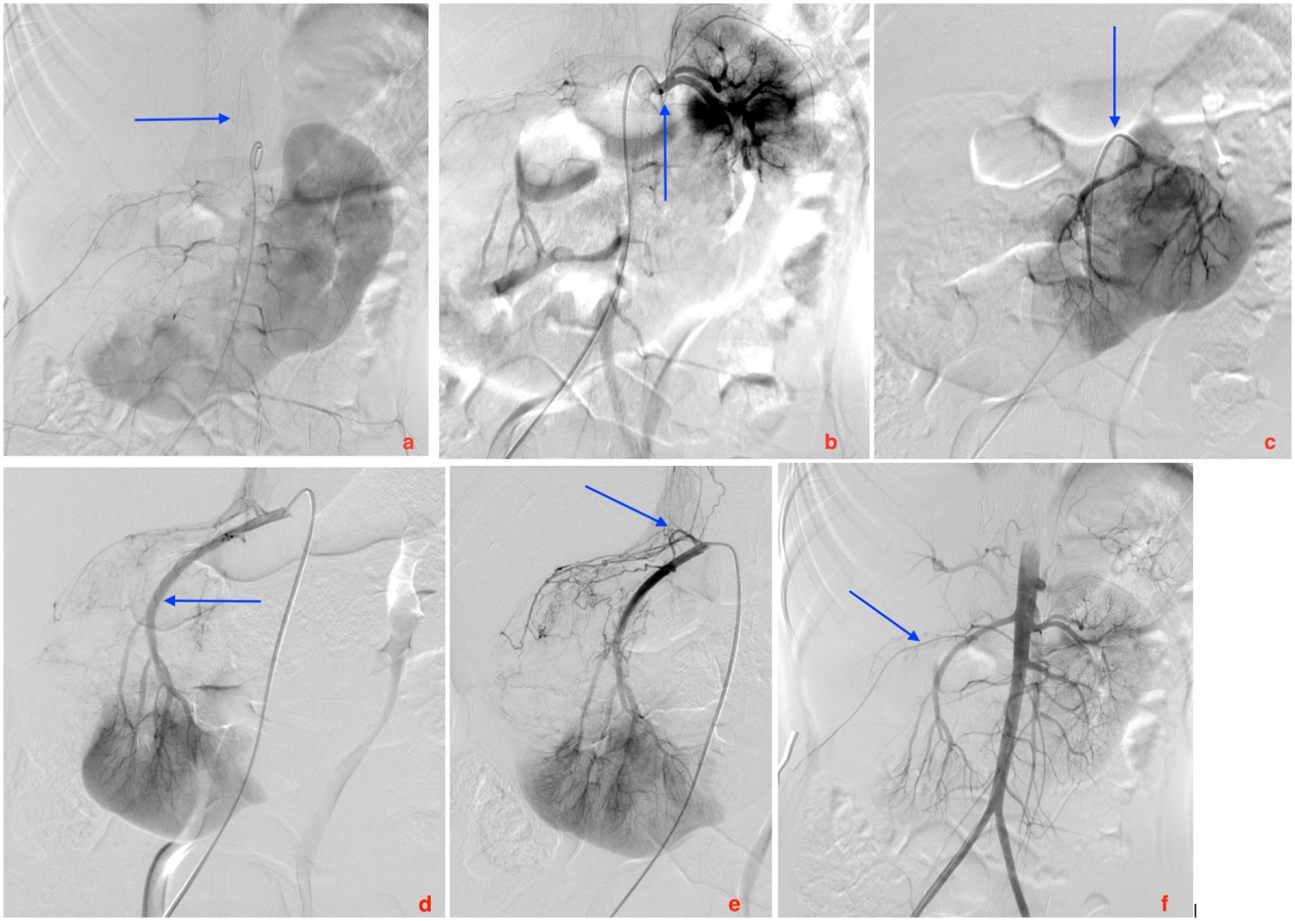
Images from the preoperative renal angiography. **(a)** Posterior anterior (PA) projection of the Adamkiewicz artery arising from metameric anastomotic circulation. **(b)** PA projection of selective angiography of left superior kidney arterial vascularization. Two superior pole arteries are detected. **(c)** PA projection of selective angiography of left middle kidney arterial vascularization **(d)** Right anterior oblique (RAO) projection of selective angiography of right infero-medial kidney arterial vascularization. **(e)** RAO projection of selective angiography of right infero-medial kidney arterial vascularization, showing a small proximal arterial vessel vascularizes the tumor of the right upper pole. **(f)** PA projection of aortography showing the whole vascularization of the horseshoe kidney, partially sparing the right upper pole, site of tumor.

The patient underwent an open partial right nephrectomy via a supraumbilical right transverse laparotomy. The HSK and the associated mass were exposed (Figure 3). The structures of the renal pedicle were carefully identified, exposed, and isolated. The tumoral vessels were identified, suture-ligated, and divided. The mass was resected with a sufficient rim of normal parenchyma, resulting in a successful NSS. The surgery was complicated by minor tumor spillage. The pathology showed a regressive post-treatment intermediate-risk nephroblastoma (stage-III according to UMBRELLA). The patient was discharged on the 10th post-operative day. The girl was then treated with adjuvant chemotherapy (vincristine, actinomycin D and doxorubicin) and radiotherapy (14.4-Gy) according to the UMBRELLA protocol.

The follow-up period until submission, which lasted 18 months, was uneventful. The girl is off-therapy and in good health.

## 3 Discussion

The variable anatomy of the HSK presents challenges for surgical approaches (2). Sato et al. described the operation on HSK as “minefield” (6). HSK exhibits variability in arterial blood supply regarding vessel size, quantity, and origin. Injury of the arterial branches causes ischemia and loss of function (2). WT arising from a HSK (1) mandates a detailed preoperative study. However, the evidence regarding the use of PORA is extremely limited (3, 9–14). Largely used in trauma management and transplantation (15) (especially in adults), PORA is rarely adopted in pediatric oncology. In extremely selected patients, it may represent a helpful adjunctive tool to guide the surgical approach, limiting complications. Furthermore, arteriography can provide images for diagnostic and treatment, such embolization of the vascular supply, as reported by many authors (mostly in adults) (3, 14). Imaging of the renal arteries may be performed using alternative, less invasive techniques (CT/MRI) because several imaging techniques have been developed in recent years to precisely depict renal vascular anatomy, with a low rate of complications. Multiple methodologies exist for examining these patients. Nonetheless, inter-institutional procedures vary significantly. Our facility has an important volume of pediatric interventional radiology, with a dedicated center even for vascular radiology even in newborn and infants. We cooperate on intricate instances for diagnostic and therapeutic objectives. A WT resulting from HSK is an unusual and hazardous situation. We believe that angiography could improve the preoperative evaluation and surgical strategy for these patients. This indication to angiography is even stronger when an embolization is needed. In order to ascertain which is more sensitive, it is necessary to perform a more comprehensive comparison between CT/MRI and PORA. In our opinion, the CT was unable to delineate the specific vascular anatomy of the tumor in the HSK in cases like the child we reported. We believe that in selected cases, PORA may be helpful as an additional complementary tool to clarify the preoperative assessment and define surgical planning. No other vessels needed to addressed at the time of surgery. In fact, the HSK's terminal arterial vascularization adds an additional risk. In complex cases like a tumor arising from an HSK, a nephron-sparing approach is essential if it is oncologically safe and surgically feasible. In our case, PORA was essential to the definition of the preoperative feasibility and safety of the nephron-sparing approach.

A literature review was conducted on this topic. (Table 1). Seven studies were identified that report the use of PORA in HSK with WT (3, 9–14). All the studies are case reports. The presentation of the WT in HSK was typical. At diagnosis, 3 patients were stage I, 2 stage II, 2 stage III, and 1 stage IV. No patients presented anaplasia. The diagnosis of abdominal mass was made by preoperative imaging in all cases, except one. In the older of the present review PORA led to a first diagnosis HSK. WT was noted at urgent abdominal exploration for acute tumor bleeding. Nevertheless, these findings are derived from a paper that was published in 1974, the oldest of the review, which is more than 50 years old. The differences in practices can be ascribed to the diverse era of the paper.

**Table 1:** Clinical and surgical characteristics of the eight patients, including ours, with WT in HSK who underwent POSA.

Author Year	Stage	Location	Other imaging	PORA details	Surgery	NSS	Complications	Recurrence
Shashikumar et al. (9) 1974	II	Right hilum	IVP	Preoperative diagnosis of HSK, but WT missed: intraoperative diagnosis.	Right nephrectomy and isthmectomy	No	No	No
Gay et al. (10) 1983	I	Isthmus	US, IVP, CT	Two branches of iliac arteries on either side supplied blood to the part of the isthmus that contained tumor.	Bilat. lower hemi nephrectomy and isthmectomy	Yes	No	No
Nesse et al. (11) 1984	I	Right lobe	IVP, CT	Stretched renal artery with the superior mesenteric artery displaced to the left. Vascular connection to tumor tissue between displaced aorta and right kidney	Right nephrectomy and isthmectomy	No	No	No
Trulock et al. (12) 1985	II	Isthmus	IVP, CT	Arterial supply from the aorta and iliac vessels.	Bilat. lower hemi nephrectomy and isthmectomy	Yes	No	No
Beseghi et al. (13) 1988	I	Right lower pole and isthmus	IVP, US	Two renal arteries, two bilateral polar arteries from aorta, and small artery from left iliac artery. Left polar artery dislocated superiorly by a round mass.	Right lower heminephrectomy and isthmectomy	Yes	No	No
Stratton et al. (14) 1994	III	Isthmus	US, CT	Unknown. Tumor embolization.	Tumorectomy	Yes	No	No
Huang et al. (3) 2004	III	Right lobe	US CT	From inferior mesenteric artery. Tumor embolization.	Right nephrectomy and partial isthmectomy	Yes	Bowel obstruction	No
Current case	IV	Right lower pole	US, CT.	One renal artery on the right and two on the left. A right branch caudal to the right renal artery.	Right partial nephrectomy	Yes	Tumor spillage	No

WT, wilms tumor; HSK, horseshoe kidney; PORA, pre-operative renal angiography; IVP, intravenous pyelogram; US, ultrasound; CT, computed tomography; NSS, nephron sparing surgery.

In seven cases (87%), PORA was successful in characterizing the arterial supply. In two cases (25%), the tumor vessels were even embolized preoperatively (3, 14). In six patients (75%), a successful NSS was possible.

RTSG-SIOP recently created a classification system for NSS (16). However, it does not specifically address the NSS criterias HSK. In the lack of a specific definition of NSS in pediatric HSK, we recently elaborated and proposed a new definition to fill the gap (7), that follows the principles of the RTSG-SIOP classification system (16). Four criteria were considered by the RTSG-SIOP when classifying NSS: (1) Surgical technique: (a) NSS (A) = partial nephrectomy = resection with a rim of normal parenchyma; (b) NSS (B) = enucleation resection without a rim of normal parenchyma; (2) Surgical resection margin (SRM): (a) Intact pseudo-capsule = (0), (b) Doubt = (1), (c) Tumor breach = (2), (3) Pathological resection margin (PRM): (a) Safe rim of renal parenchyma on resection margin, except nephroblastomatosis = (0), (b) Intact pseudo-capsule along the resection margin = (1), (c) Tumor breach = (2), (4). Remaining renal parenchyma (RRP). RTSG-SIOP suggests reporting each case as follows: NSS(X)–SRM (n)-PRM(n)-RRP(*n*%).

Instead, based on the classification by Godzinski (16), we elaborated a definition of NSS in HSK. NSS is the procedure in which resection of tumor with a rim of normal renal parenchyma was performed [NSS(A) and PRM (0)], with an intact pseudo-capsule [SRM (0)] and >50% of RRP (considering 50% as one renal lobe and half of the isthmus). In tumors arising from the isthmus, more than 50% of each lobe should be preserved.

The present study has limitations. This is a case report with a literature review. Is noteworthy, that not all institutions have access to pediatric angiography, because it requires dedicated technologies and radiologists. Finally, it was not possible to compare PORA with other diagnostic techniques to identify differences in sensitivity and specificity. Future investigations are needed to thoroughly analyze this aspect, particularly in pediatric populations, which is currently lacking in data.

## 4 Conclusion

PORA seemed safe and useful for NSS in WT arising from HSK. Despite its invasiveness, this study may have an adjunctive role in the extremely selected patients.

## Data Availability

The original contributions presented in the study are included in the article/Supplementary Material, further inquiries can be directed to the corresponding author.
